# Supplementary material for: Aggregative trans-eQTL analysis detects trait-specific target gene sets in whole blood
Source: Nat Commun. 2022 Jul 26;13:4323. doi: 10.1038/s41467-022-31845-9 (PMC9325868; doi:10.1038/s41467-022-31845-9)
Supplement: Supplementary file 3 — Description of Additional Supplementary Files [file 41467_2022_31845_MOESM3_ESM.pdf]

File Name: Supplementary Data 1
Description: Results from type-1 error simulation under competitive null hypothesis and power simulation under global null hypothesis under the causal models shown in Figure 2B-C

File Name: Supplementary Data 2
Description: Selected variants (rsID, chromosome and position on GRCh 38) and genes from ARCHIE components for SCZ, UC and PC

File Name: Supplementary Data 3
Description: Enriched Pathways for target genes selected by ARCHIE for SCZ

File Name: Supplementary Data 4
Description: Enrichment of selected SCZ target genes from ARCHIE analysis among targets of TFs curated from different databases

File Name: Supplementary Data 5
Description: Enriched Pathways for target genes selected by ARCHIE component 1 for UC

File Name: Supplementary Data 6
Description: Enriched Pathways for target genes selected by ARCHIE component 2 for UC

File Name: Supplementary Data 7
Description: Enrichment of selected UC target genes from ARCHIE analysis among targets of TFs curated from different databases

File Name: Supplementary Data 8
Description: Enriched Pathways for target genes selected by ARCHIE component 1 for PC

File Name: Supplementary Data 9
Description: Enriched Pathways for target genes selected by ARCHIE component 2 for PC

File Name: Supplementary Data 10
Description: Enrichment of selected PC target genes from ARCHIE analysis among targets of TFs curated from different databases

File Name: data.zip
Description: zipped folder containing source data for the figures. The individual files within are marked by the name of the Figure and panel they are plotted in.

Contents within data.zip:

File Name: 3A.txt
Description: Source data for Figure 3A. Each row represents a resampling iteration for the competitive null for Schizophrenia (SCZ). The final row represents the observed values for SCZ (red dots in the figure).

File Name: 4A.txt
Description: Source data for Figure 4A. Each row represents a resampling iteration for the competitive null for Ulcerative Colitis (UC). The final row represents the observed values for UC (red dots in the figure).

File Name: 5A.txt
Description: Source data for Figure 5A. Each row represents a resampling iteration for the competitive null for Prostate Cancer (PC). The final row represents the observed values for PC (red dots in the figure).

File Name: 3D.txt
Description: Source data for Figure 3D. Categories A and B represent the data for “trans-heritability estimated with selected SNPs and random genes” and “trans-heritability estimated with selected SNPs and genes on

random trait” respectively. The second column represents the values used for plotting the violin plot in each of the categories. The third column (constant value) represents the estimated trans-heritability for SCZ with the selected SNPs and genes.

File Name: 4D.txt
Description: Source data for Figure 4D. Categories A and B represent the data for “trans-heritability estimated with selected SNPs and random genes” and “trans-heritability estimated with selected SNPs and genes on random trait” respectively. The second column represents the values used for plotting the violin plot in each of the categories. The third column (constant value) represents the estimated trans-heritability for UC with the selected SNPs and genes.

File Name: 5D.txt
Description: Source data for Figure 5D. Categories A and B represent the data for “trans-heritability estimated with selected SNPs and random genes” and “trans-heritability estimated with selected SNPs and genes on random trait” respectively. The second column represents the values used for plotting the violin plot in each of the categories. The third column (constant value) represents the estimated trans-heritability for PC with the selected SNPs and genes.

File Name: 3B.txt
Description: Source data for Figure 3B. Z-values for the standard trans-eQTL association mapping reported by eQTLGen consortium for the selected SNPs and genes for the significant ARCHIE component of SCZ. Each row represents a selected SNP, and each column represents a selected gene in the ARCHIE component.

File Name: 5B.txt
Description: Source data for Figure 5B. p-values for the standard trans-eQTL association mapping reported by eQTLGen consortium for the selected SNPs and genes for the significant ARCHIE component 1 of PC.

Each row represents a selected SNP, and each column represents a selected gene in the ARCHIE component 1.

File Name: 5C.txt
Description: Source data for Figure 5C. p-values for the standard trans-eQTL association mapping reported by eQTLGen consortium for the selected SNPs and genes for the significant ARCHIE component 2 of PC. Each row represents a selected SNP, and each column represents a selected gene in the ARCHIE component.
