# Supplementary material for: Aggregative trans-eQTL analysis detects trait-specific target gene sets in whole blood
Source: Nat Commun. 2022 Jul 26;13:4323. doi: 10.1038/s41467-022-31845-9 (PMC9325868; doi:10.1038/s41467-022-31845-9)
Supplement: Supplementary file 5 — Reporting Summary [file 41467_2022_31845_MOESM5_ESM.pdf]

nature research

Corresponding author(s): Nilanjan Chatterjee

Last updated by author(s): May 9, 2022

Reporting Summary

Nature Research wishes to improve the reproducibility of the work that we publish. This form provides structure for consistency and transparency in reporting. For further information on Nature Research policies, see our Editorial Policies and the Editorial Policy Checklist.

Statistics

For all statistical analyses, confirm that the following items are present in the figure legend, table legend, main text, or Methods section.

n/a	Confirmed	
☐	☒	The exact sample size (*n*) for each experimental group/condition, given as a discrete number and unit of measurement
☒	☐	A statement on whether measurements were taken from distinct samples or whether the same sample was measured repeatedly
☐	☒	The statistical test(s) used AND whether they are one- or two-sided<br>*Only common tests should be described solely by name; describe more complex techniques in the Methods section.*
☐	☒	A description of all covariates tested
☐	☒	A description of any assumptions or corrections, such as tests of normality and adjustment for multiple comparisons
☐	☒	A full description of the statistical parameters including central tendency (e.g. means) or other basic estimates (e.g. regression coefficient) AND variation (e.g. standard deviation) or associated estimates of uncertainty (e.g. confidence intervals)
☐	☒	For null hypothesis testing, the test statistic (e.g. *F*, *t*, *r*) with confidence intervals, effect sizes, degrees of freedom and *P* value noted<br>*Give P values as exact values whenever suitable.*
☒	☐	For Bayesian analysis, information on the choice of priors and Markov chain Monte Carlo settings
☒	☐	For hierarchical and complex designs, identification of the appropriate level for tests and full reporting of outcomes
☒	☐	Estimates of effect sizes (e.g. Cohen's *d*, Pearson's *r*), indicating how they were calculated

Our web collection on statistics for biologists contains articles on many of the points above.

Software and code

Policy information about availability of computer code

Data collection	The primary source of the data is the publicly available summary statistics found at https://www.eqtlgen.org/ (downloaded on 10/01/2019). No additional software were used to collect the data. Additional publicly accessible and/or previously published data sources are mentioned in the Data availability Section.
Data analysis	eQTLGen (trans-eQTL summary statistics): https://www.eqtlgen.org/trans-eqtls.html<br>GTEx: https://www.gtexportal.org/home/<br>1000 Genomes: https://www.internationalgenome.org/data/<br>UKBiobank: https://www.ukbiobank.ac.uk/<br>FUMA: https://fuma.ctglab.nl/<br>ShinyGO v0.65: http://bioinformatics.sdstate.edu/go/<br>STRING v11.0: https://string-db.org/cgi/<br>The main software for the method has been made available through GitHub at https://github.com/diptavo/ARCHIE (initial release). This repository will be continuously updated with further modifications to the code.<br>GitHub: https://github.com/diptavo/ARCHIE (initial release)<br>Additionally we have linked the GitHub repository to a zenodo repository:<br>Zenodo: https://doi.org/10.5281/zenodo.6533207

For manuscripts utilizing custom algorithms or software that are central to the research but not yet described in published literature, software must be made available to editors and reviewers. We strongly encourage code deposition in a community repository (e.g. GitHub). See the Nature Research guidelines for submitting code & software for further information.

Data

Policy information about availability of data

All manuscripts must include a data availability statement. This statement should provide the following information, where applicable:

- Accession codes, unique identifiers, or web links for publicly available datasets
- A list of figures that have associated raw data
- A description of any restrictions on data availability

The primary source of the data is the publicly available summary statistics found at https://www.eqtlgen.org/.
Further, additional individual data from UK Biobank was used under application 17712 and from Genotype Tissue Expression consortium (GTEx) with dbGAP Accession ID phs000424.v8.p2.
Individual level data for the DGN cohort is available by application through the NIMH Center for Collaborative Genomic Studies on Mental Disorders. Instructions for requesting access to data can be found at https://www.nimhgenetics.org/access_data_biomaterial.php, and inquiries should reference the "Depression Genes and Networks study (D. Levinson, PI)". The information has been mentioned in the Data Availability section. The procedure to access individual level data from the website has been detailed in the following webpage: https://www.nimhgenetics.org/request-access/how-to-request-access. The access is controlled due to presence of individual level data. Proper approval and requests as outlined in the above page should be followed and would be reviewed by trans-NIH Data Access Committee to grant permission to use individual level data.

Field-specific reporting

Please select the one below that is the best fit for your research. If you are not sure, read the appropriate sections before making your selection.

☒ Life sciences ☐ Behavioural & social sciences ☐ Ecological, evolutionary & environmental sciences

For a reference copy of the document with all sections, see nature.com/documents/nr-reporting-summary-flat.pdf

Life sciences study design

All studies must disclose on these points even when the disclosure is negative.

Sample size	We analyzed publicly available trans-eQTL summary statistics from eQTLGen consortium. A detailed description of sample size (31,684) is mentioned in https://www.eqtlgen.org/ and in the preprint https://doi.org/10.1101/447367<br>We analyzed the data made available by the consortium and hence did not have explicit control over determining the sample size. However, this the largest till date transcriptomic study in Whole Blood in terms of sample size and hence should have substantially more power to identify association between variants and gene-expressions.
Data exclusions	We analyzed publicly available trans-eQTL summary statistics from eQTLGen consortium. A detailed description of data processing criteria is outlined in the preprint https://doi.org/10.1101/447367.<br>We did not systematically exclude any data from the analysis.<br>We analyzed the traits which had at least 100 genetic variants (SNPs) reported in the database. Further, for a given trait, we excluded the summary statistics for the genes which were less than 5Mb away from at least one SNP for the trait in the database. The choice of this study design has been also detailed in the Results section.
Replication	Not applicable.<br>The analysis described in the manuscript is a secondary analysis for the summary data published by the eQTLGen consortium. We have not carried out any replication analysis of the identified genes due to the lack of availability of similar transcriptomic studies with sufficient statistical power. Instead, we have demonstrated several independent lines of evidence to establish the relevance of the identified gene-sets for the diseases/phenotypes being analyzed.
Randomization	Not Applicable<br>Since we are not directly testing for causal relationship, standard randomization is not applicable in this case. However, in the testing for significance section (in Methods), we have described a randomization and resampling procedure to evaluate the significance of the identified gene-sets.
Blinding	Not Applicable<br>Since we only analyze summary statistics in this case and the setup is not a classical treatment-control design, blinding does not apply in the analysis.

Reporting for specific materials, systems and methods

We require information from authors about some types of materials, experimental systems and methods used in many studies. Here, indicate whether each material, system or method listed is relevant to your study. If you are not sure if a list item applies to your research, read the appropriate section before selecting a response.

Materials & experimental systems

n/a	Involved in the study
☒	☐ Antibodies
☒	☐ Eukaryotic cell lines
☒	☐ Palaeontology and archaeology
☒	☐ Animals and other organisms
☒	☐ Human research participants
☒	☐ Clinical data
☒	☐ Dual use research of concern

Methods

n/a	Involved in the study
☒	☐ ChIP-seq
☒	☐ Flow cytometry
☒	☐ MRI-based neuroimaging
